# Delayed Diagnoses of Cardiac Amyloidosis

**DOI:** 10.1016/j.jaccas.2025.103910

**Published:** 2025-07-09

**Authors:** Cooper B. Kersey, Graham Bevan, Douglas Leedy, Marta Alhama-Belotto, Babak Nazer, Kelly Smith, Ron Blankstein, Mathew S. Mauer, Richard K. Cheng

**Affiliations:** a Department of Medicine, Division of Cardiology, University of Washington, Seattle, Washington, USA; b Department of Pathology, University of Washington, Seattle, Washington, USA; c Department of Medicine, Division of Cardiology, Brigham and Women’s Hospital, Boston, Massachusetts, USA; d Department of Medicine, Division of Cardiology, Columbia University, New York, New York, USA

**Keywords:** cardiac amyloidosis, cardiac sarcoidosis, case report, multimodality imaging, nonischemic cardiomyopathy

## Abstract

Cardiac sarcoidosis (CS) can be challenging to accurately diagnose and relies on a complex diagnostic framework because of the limited sensitivity of endomyocardial biopsy. Frequently, the diagnosis of CS depends on cardiac ^18^F-fluorodeoxyglucose positron emission tomography, which has limited specificity for sarcoidosis. We report 3 cases of individuals who were initially diagnosed and treated for isolated CS based on multimodality cardiac imaging and later definitively reclassified to wild-type transthyretin cardiac amyloidosis from histopathology obtained by endomyocardial biopsy.

## Graphical Abstract

**Figure:**
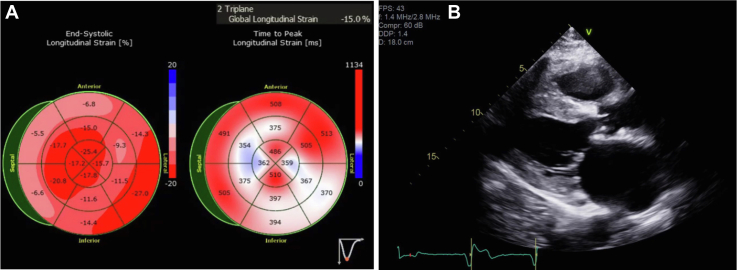

The noninvasive diagnosis of cardiac sarcoidosis (CS) is challenging and requires integrating a patient’s clinical presentation with multimodality imaging data. The incidence of CS is increasing, partially driven by increased awareness.^1^ In the absence of an endomyocardial biopsy demonstrating noncaseating granulomas, both the Japanese Cardiovascular Society (JCS) and Heart Rhythm Society guidelines recommend using a combination of clinical, electrocardiographic, and advanced imaging criteria to diagnose CS.^2^ The patchy nature of myocardial involvement in CS limits the sensitivity of endomyocardial biopsy for detecting sarcoidosis (20%-30%), and as a result, there is frequently a dependence on multimodality imaging to ascertain the diagnosis of CS.^3^ Cardiac ^18^F-fluorodeoxyglucose positron emission tomography (^18^F FDG PET) is included in the diagnostic algorithm for CS because of its ability to detect myocardial inflammation with high sensitivity.^3^ However, the specificity of ^18^F FDG PET for CS may only be 33% because of FDG avidity of other cardiomyopathies.^3^^,^^4^ We report 3 patients who were diagnosed and treated for CS based on ^18^F FDG PET, despite clinical and echocardiographic features consistent with cardiac amyloidosis, before being reclassified as wild-type transthyretin cardiac amyloidosis (ATTR-CA) with endomyocardial biopsy.

**Take-Home Messages**

• Cardiac sarcoidosis is a challenging diagnosis to make and needs to be considered within a continuum of likelihood.
• There is radiographic overlap between many nonischemic cardiomyopathies on ^18^F FDG PET.
• When diagnostic uncertainty is present in the workup of a nonischemic cardiomyopathy, histologic confirmation and genetic testing should be pursued.

## Case Reports

**Figure 1:**
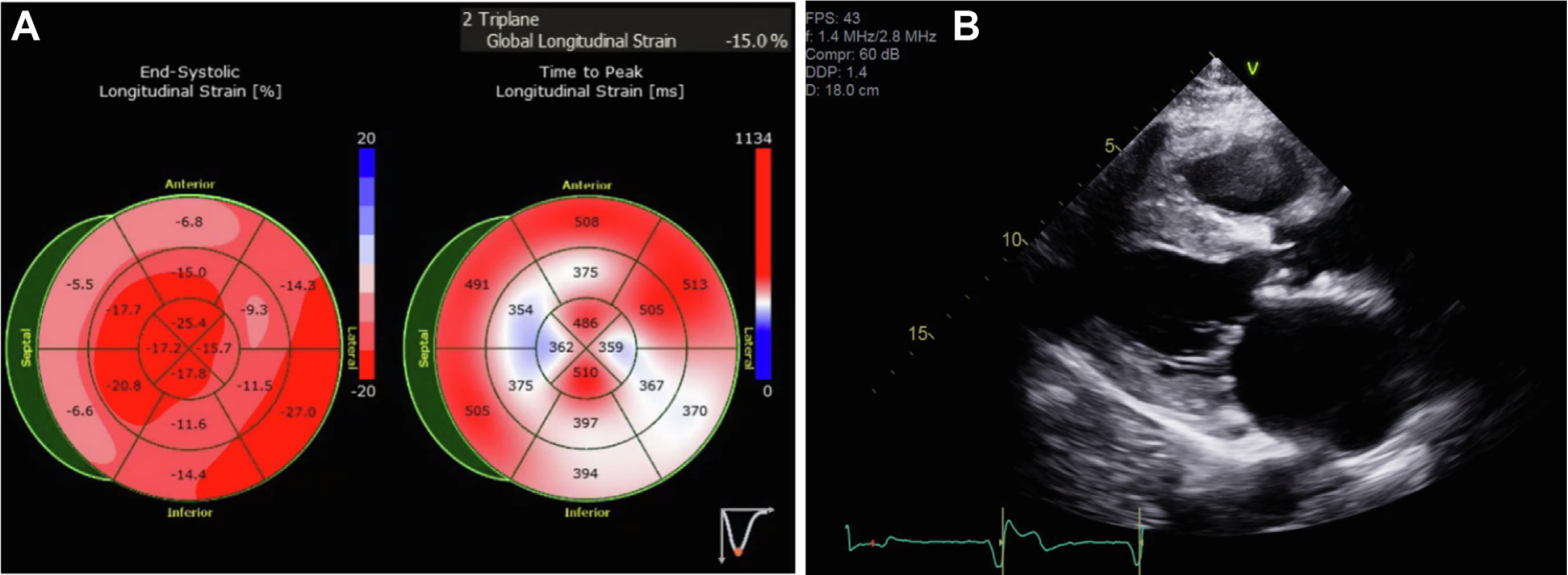
Echocardiogram Images for Patient 1 (A) Patient 1 had reduced global longitudinal strain on the echocardiogram. (B) Transthoracic echocardiogram demonstrating mild symmetrical increase in left ventricular wall thickness (1.3 cm) for patient 1.

**Figure 2:**
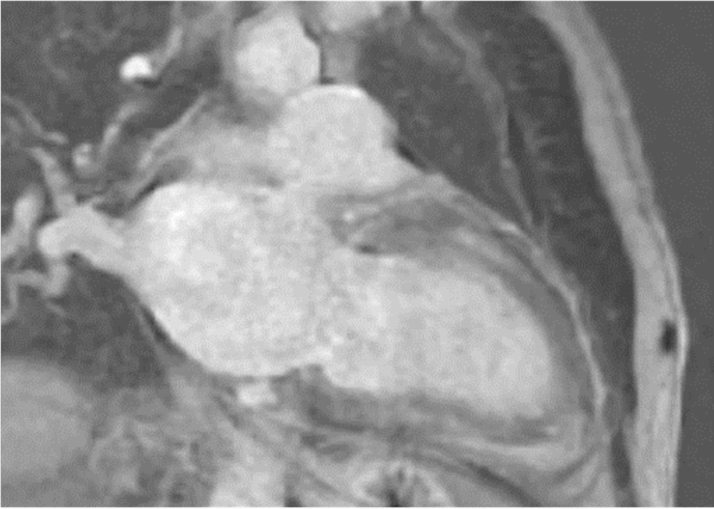
Cardiac Magnetic Resonance Imaging of Patient 1 Showing Patchy Late Gadolinium Enhancement

Patient 1, a 70-year-old man with lambda smoldering myeloma (on revlimid-dexamethasone), paroxysmal atrial fibrillation, tachybrady syndrome, and a nonischemic cardiomyopathy, was referred for a second opinion. He had previously undergone a coronary angiogram that demonstrated no obstructive coronary artery disease. An electrocardiogram revealed that he was in atrial fibrillation with normal voltage. His transthoracic echocardiogram showed an increase in left ventricular wall thickness (1.5 cm), left ventricular ejection fraction of 58%, severe biatrial enlargement, and reduced global longitudinal strain (–15%) (Figures 1A and 1B, Video 1). A cardiac magnetic resonance imaging (CMR) was notable for patchy, predominantly midwall, late gadolinium enhancement (LGE) in the anterior, anterolateral, inferolateral, and septal walls (Figure 2). He subsequently underwent a cardiac ^18^F FDG PET that showed intense focal FDG uptake in the basal anteroseptal wall as well as FDG uptake in the lateral wall with matched perfusion defects. He was given the presumed diagnosis of CS and started on prednisone 40 mg daily. On serial ^18^F FDG PET 6 months later, there was resolution of FDG uptake. Suspicion for CS in his case was based on corticosteroid responsiveness, patchy LGE on CMR, and the ^18^F FDG PET findings. His prednisone was initially tapered but then restarted because of nonsustained ventricular tachycardia seen on device monitoring. Repeat ^18^F FDG PETs showed increased FDG avidity that did not dissipate with continued immunosuppression. He was referred for a second opinion and because of diagnostic uncertainty, he underwent an endomyocardial biopsy that was Congo red positive on histology. The sample was sent for liquid chromatography mass spectrometry, which detected a peptide profile consistent with transthyretin type amyloid deposition. The patient had simultaneously undergone genetic testing that showed no pathogenic variants in the transthyretin gene, and he was reclassified as having wild-type ATTR-CA. He was subsequently started on tafamidis, a transthyretin tetramer stabilizer, and tapered off immunosuppression.

**Figure 3:**
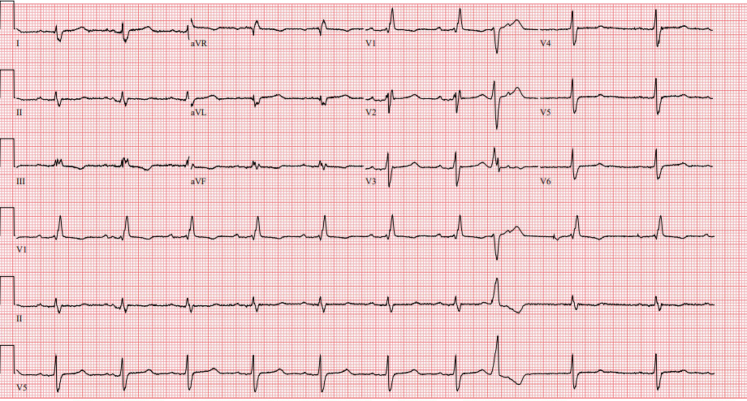
Electrocardiogram of Patient 2 Showing First-Degree Atrioventricular Block and a Right Bundle Branch Block

**Figure 4:**
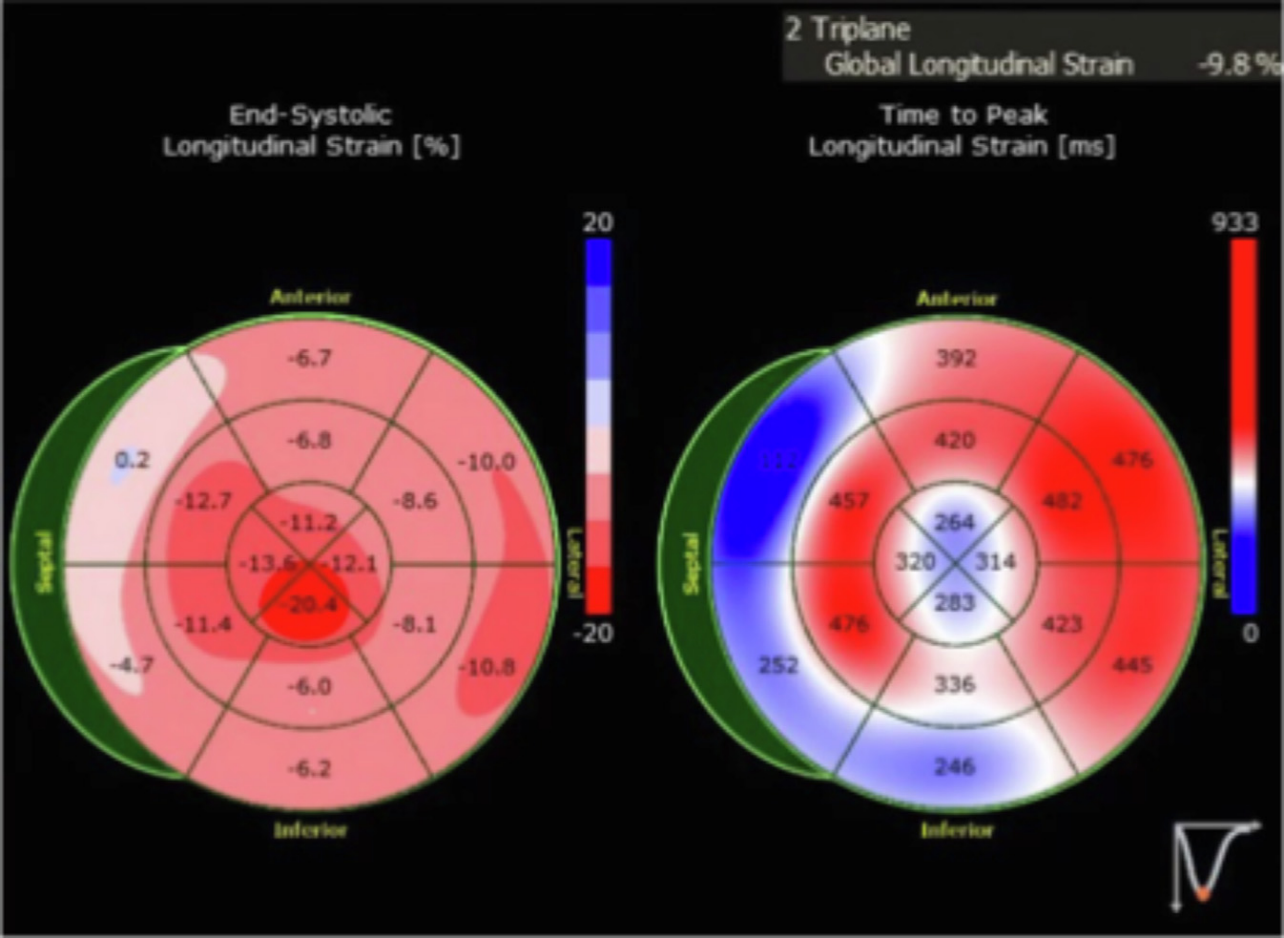
Patient 2 Had Reduced Global Longitudinal Strain on the Echocardiogram With Apical Sparing

**Figure 5:**
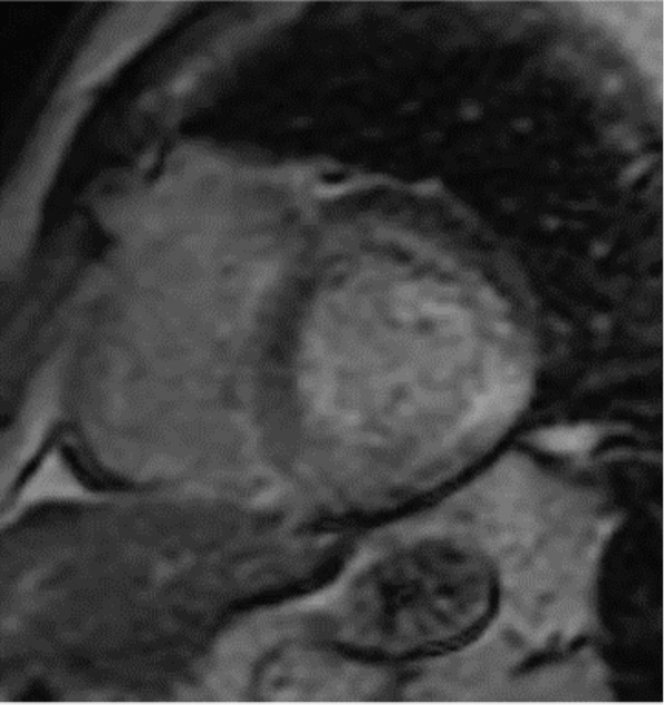
Cardiac Magnetic Resonance Imaging of Patient 2 Showing Late Gadolinium Enhancement of the Basal Midinferolateral, Basal Midlateral, and Basal Inferior Wall

Patient 2, a 60-year-old man with a history of paroxysmal atrial fibrillation, nonsustained ventricular tachycardia, bilateral carpal tunnel syndrome, and spinal stenosis, was referred for a second opinion regarding the etiology of his cardiomyopathy. An electrocardiogram at the time of his referral demonstrated sinus rhythm with first-degree atrioventricular block (PR interval 242 ms), normal voltage, and a right bundle branch block (Figure 3). His echocardiogram revealed a severe increase in left ventricular wall thickness (2.2 cm), a left ventricular ejection fraction of 60%, and reduced global longitudinal strain with apical sparing pattern (Figure 4, Video 1). A coronary angiogram showed no evidence of obstructive coronary artery disease. CMR showed focal late gadolinium in the basal midinferolateral, basal midlateral, and basal inferior walls (Figure 5, Video 2). The distribution of CMR findings raised concern for Fabry’s disease, and he was referred for genetic testing with a comprehensive cardiomyopathy panel. Genetic testing did not reveal any pathogenic mutations associated with Fabry’s disease or hypertrophic cardiomyopathy. He subsequently underwent cardiac ^18^F FDG PET that showed mild FDG uptake in the basal midinferolateral wall and midanteroseptal wall with matched perfusion defects. Clinical suspicion for CS was driven by his unexplained nonsustained ventricular tachycardia, abnormal LGE on CMR, and ^18^F FDG PET findings. Immunosuppressive therapy was initiated with prednisone and methotrexate for a presumed diagnosis of CS with serial ^18^F FDG PET demonstrating persistent regional FDG uptake. He was referred for a second opinion for refractory CS after an extended course of immunosuppression. Because of diagnostic uncertainly of CS from a lack of response to immunosuppression, he underwent an endomyocardial biopsy that stained positive for Congo red. Mass spectrometry confirmed transthyretin amyloid deposition. A comprehensive genetic cardiomyopathy panel was repeated but did not reveal pathogenic variants in the transthyretin gene and he was diagnosed with wild-type ATTR-CA. The patient was subsequently enrolled in a clinical trial for a transthyretin knockdown agent, started on tafamidis, and his immunosuppression was stopped.

**Figure 6:**
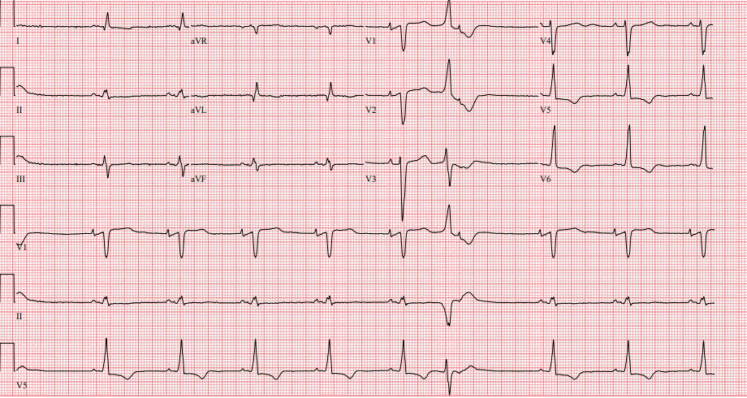
Electrocardiogram of Patient 3 Showing Low Voltage in Limb Leads With Pseudoinfarct Pattern

**Figure 7:**
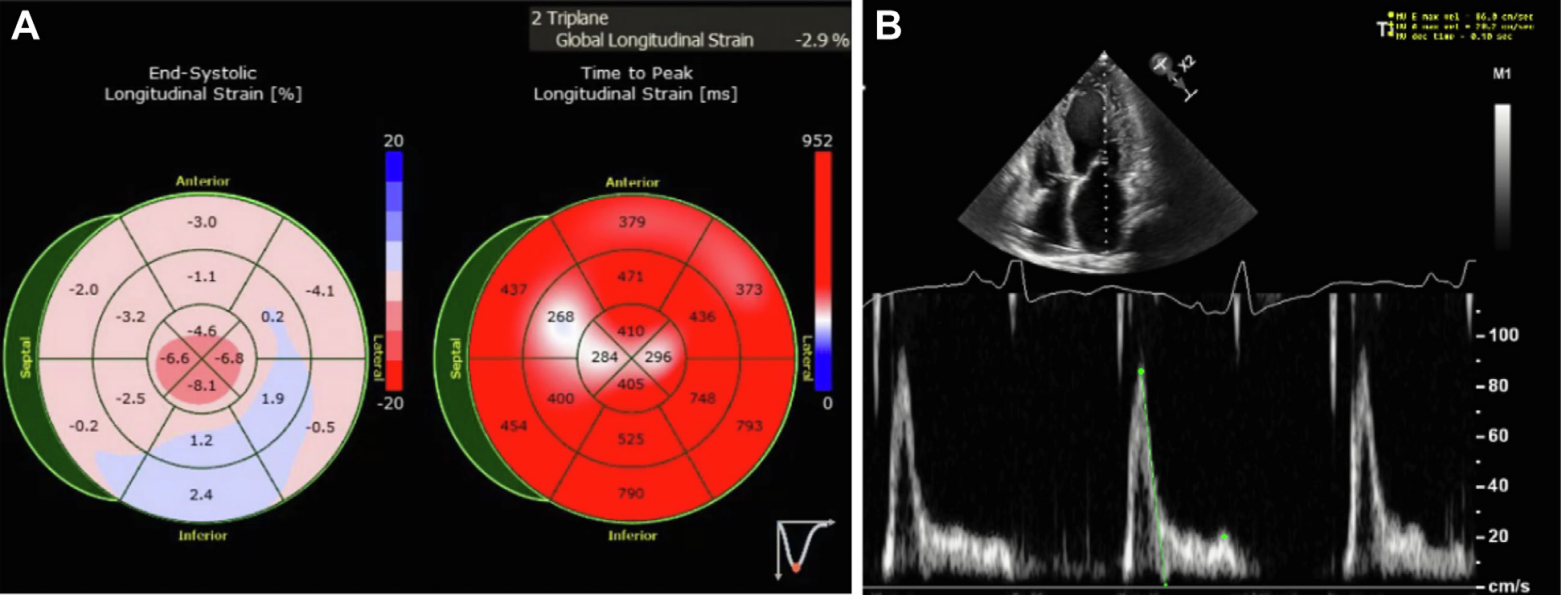
Echocardiogram Images for Patient 3 (A) Patient 3 had reduced global longitudinal strain with apical sparing pattern on transthoracic echocardiogram. (B) Mitral inflow Doppler signal consistent with restrictive filling pattern for patient 3.

**Figure 8:**
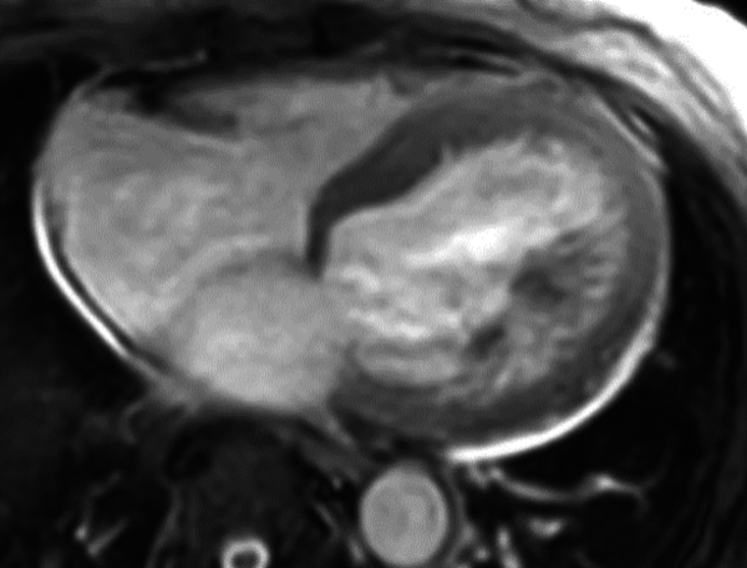
Cardiac Magnetic Resonance Imaging for Patient 3 Showing Patchy Late Gadolinium Enhancement

**Figure 9:**
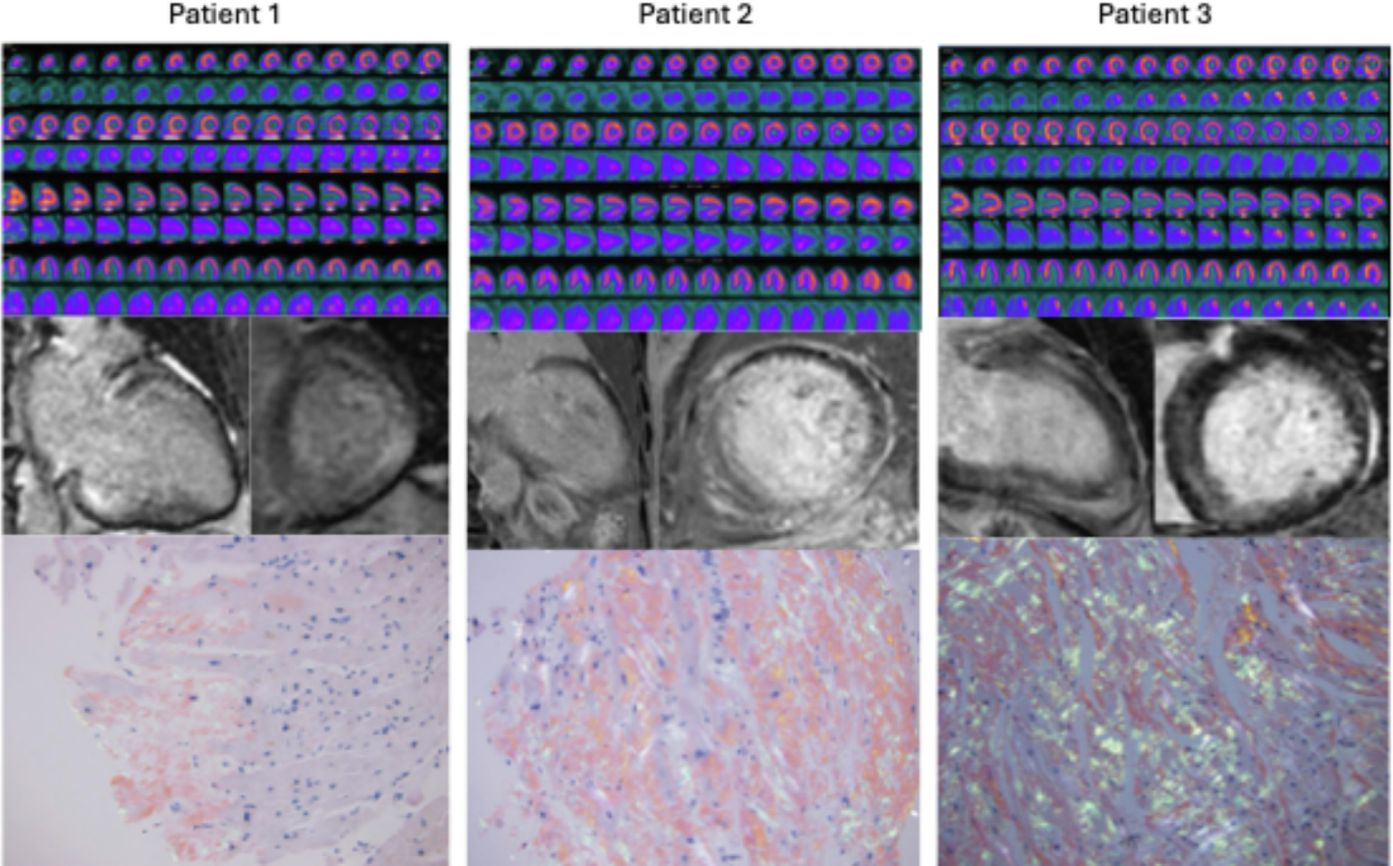
FDG PET Images, Cardiac Magnetic Resonance Images, and Histology for Patients 1 to 3 ^18^F-fluorodeoxyglucose positron emission tomography (^18^F FDG PET) images, cardiac magnetic resonance (CMR) images, and histology slides for patients 1 to 3 (left to right). ^18^F FDG PET images show FDG uptake, CMR images demonstrate delayed gadolinium enhancement, and histology with Congo red stain reveals amyloid deposition.

Patient 3, a 77-year-old woman with a history of a nonischemic cardiomyopathy with a primary prevention defibrillator in place, high premature ventricular contraction burden, and paroxysmal atrial fibrillation, was undergoing workup for the etiology of her cardiomyopathy. An electrocardiogram demonstrated sinus rhythm with low voltage in the limb leads and occasional premature ventricular contractions (Figure 6). A transthoracic echocardiogram showed a moderately dilated left ventricle with a mild increase in left ventricular wall thickness, severely decreased left ventricular ejection fraction (23%), panvalvular thickening, severely reduced global longitudinal strain imaging with apical sparing, and a restrictive filling pattern (Figures 7A and 7B, Videos 3a and 3b). A coronary angiogram obtained previously demonstrated no obstructive coronary artery disease. Free light chains to screen for light chain amyloidosis did not reveal a monoclonal gammopathy. A CMR demonstrated patchy midmyocardial LGE of the interventricular septum, anterior, and inferior walls (Figure 8, Video 4). She then underwent a cardiac ^18^F FDG PET that revealed a small area of increased FDG avidity in the midinferolateral wall with a matched perfusion defect. There was clinical suspicion for CS because of her unexplained left ventricular dysfunction, patchy LGE on CMR, and ^18^F FDG PET findings, and she was referred for treatment for CS. However, given the diagnostic uncertainty, she underwent voltage map-guided endomyocardial biopsy before initiating treatment. The biopsy specimen was Congo red positive, and mass spectrometry confirmed transthyretin type amyloid deposition. Genetic testing revealed no pathogenic variants in the transthyretin gene. She was diagnosed with wild-type ATTR-CA and started on tafamidis. A summary of the ^18^F FDG PET images, CMR images, and histology slides for patients 1 to 3 (left to right) are shown in Figure 9.

## Discussion

Our case series adds to the existing evidence that the likelihood of CS needs to be considered within a continuum and that the diagnosis is not binary in nature.^5^ Not all ^18^F FDG avid cardiomyopathies represent CS, even when presenting within the clinical context of unexplained arrhythmias, conduction system disease, or left ventricular dysfunction. The list of cardiomyopathic processes that lead to increased myocardial ^18^F FDG uptake on PET is growing and includes myocarditis, genetic cardiomyopathies (hypertrophic cardiomyopathy, Lamin, desmoplakin), and now wild-type ATTR-CA.^4^^,^^6^ The etiology of tracer uptake in ATTR-CA has been hypothesized to be due to inflammation associated with amyloid fibril deposition or direct myocardial inflammation in cases of myocarditis.^6^^,^^7^ However, there is emerging evidence that ^18^F FDG uptake on PET can be driven by altered glucose metabolism by the myocardium in genetic cardiomyopathies and other cause-specific cardiomyopathies. It is possible that mechanistically it is a combination of altered metabolism in addition to myocardial inflammation.^4^^,^^8^^,^^9^ Furthermore, incomplete myocardial glucose suppression, either because of physiological reasons (eg, inability to suppress FDG from the myocardium) or noncompliance with the prescribed ketogenic diet, can result in increased ^18^F FDG avidity in the absence of CS.^10^ Given the low specificity of ^18^F FDG PET for CS, it is imperative that the results of ^18^F FDG PETs are interpreted in the broader clinical context of the patient and that clinicians do not exhibit anchoring bias based on the results of imaging alone. To avoid premature closure when working up nonischemic cardiomyopathies, we advocate for incorporating genetic testing and endomyocardial biopsy into the diagnostic workup earlier when there is uncertainty about the root cause of a patient’s infiltrative or inflammatory cardiomyopathy.

In the cases presented, all patients had clinical and echocardiographic features suggestive of ATTR-CA but were incorrectly diagnosed with CS based solely on the result of ^18^F FDG PETs. Because of the increasing awareness of CS, it is likely that misclassification of other cardiomyopathies, such as the cases presented here, as CS based on over-reliance on imaging results exist.^5^ The patients presented here exemplify why it is imperative to interpret the results of ^18^F FDG PETs in the broader clinical context of the patient to avoid misdiagnosis. All patients presented here had some clinical features of isolated CS, but none of them met the full JCS criteria, which should have prompted further workup such as endomyocardial biopsy. Both patients 1 and 2 had nonsustained ventricular arrhythmias coupled with patchy LGE on CMR and FDG avidity on ^18^F FDG PET, which satisfies 2 major and 1 minor JCS criteria.^2^ The JCS criteria for CS have been validated for the diagnosis of isolated CS, and the stringency of the diagnostic criteria (4 of 5 major criteria required in the absence of myocardial or extracardiac histology positive for sarcoidosis) exemplifies the overlap in both the clinical presentation and advanced imaging findings of CS with other nonischemic cardiomyopathies. The final patient had severe left ventricular dysfunction, unexplained nonsustained ventricular arrhythmias, and patchy LGE on CMR and FDG avidity on ^18^F FDG PET. Satisfying 3 major and 1 minor criteria for CS, this patient also would not have met JCS criteria for CS without histology. The JCS criteria include both CMR and ^18^F FDG PET because these 2 imaging modalities offer complementary information. CMR is highly sensitive (95%) for detecting CS, and ^18^F FDG PET can detect active inflammation and be used to guide anti-inflammatory therapy.^11^ For all 3 cases presented, the patients did not satisfy the JCS criteria for CS, and further workup with endomyocardial biopsy should have been pursued before initiation of immunosuppression.

Diagnostic clarity is crucial in the space of inflammatory and infiltrative cardiomyopathies because it has therapeutic implications. An accurate diagnosis is necessary because the treatment options for CS entail initiation of immunosuppression, frequently for extended periods of time; hence, the risk for adverse effects of treatment must be balanced against the likelihood of benefit. In cases where the diagnosis is unclear, the decision on whether to start immunosuppression becomes more challenging. Further, the involvement of a multidisciplinary team of experts familiar with nuances of specific cardiomyopathies should be pursued. In this series, each case was misclassified as CS initially, and after referring to a cardiomyopathy expert, endomyocardial biopsy was pursued because of perceived diagnostic uncertainty, leading to reclassification of all 3 cases as ATTR-CA. There have been many advents in therapeutics for cardiac amyloidosis in recent years. Tafamidis, which binds to and stabilizes the transthyretin tetramer to inhibit misfolding and abrogating the formation of ATTR amyloid fibrils, was the first therapeutic for ATTR-CA approved by the U.S. Food and Drug Administration in 2019. In cases of ATTR-CA, early diagnosis is imperative because tafamidis slows disease progression but is not curative. When disease-altering therapies for alternative diagnoses are available and the risk for harm from treatment exists, the evaluation is insufficient to presume CS based on imaging alone when not all diagnostic clues point toward CS.

This case series adds to the previously reported literature on ^18^F FDG PET avidity in conditions other than CS. First, CS is a challenging diagnosis and needs to be considered within a continuum of likelihood; when some clinical clues do not fit, such as in the aforementioned cases, histologic confirmation and genetic testing should be pursued to guide treatment decisions. Second, although multimodality cardiac imaging has made many advances, radiographic overlap remains for differential cause-specific cardiomyopathies.

We present 3 cases where the individual was given a diagnosis of CS based on imaging findings and then reclassified as ATTR-CA after endomyocardial biopsy. Future studies are needed to better define which cardiomyopathies may result in ^18^F FDG PET avidity, but, more importantly, the underlying mechanistic pathways of why this occurs may potentially lead to future therapeutic targets.

## Funding Support and Author Disclosures

The authors have reported that they have no relationships relevant to the contents of this paper to disclose.

## Appendix

Video 1 Parasternal long-axis view of patient 2’s echocardiogram demonstrates a normal ejection fraction and severe increase in left ventricular wall thickness (2.2 cm).

Video 2 Cine image from the cardiac magnetic resonance imaging of patient 2 demonstrating severe increase in left ventricular wall thickness (2.2 cm).

Video 3a (a and b) Parasternal long-axis and apical 4-chamber images demonstrating a mild increase in left ventricular wall thickness and severely reduced left ventricular ejection fraction.

Video 3b (a and b) Parasternal long-axis and apical 4-chamber images demonstrating a mild increase in left ventricular wall thickness and severely reduced left ventricular ejection fraction.

Video 4 Cine image from the cardiac magnetic resonance imaging of patient 3 demonstrating severely reduced left ventricular systolic function.
